# Investigation of Heat Transfer and Pressure Drop in Microchannel Heat Sink Using Al_2_O_3_ and ZrO_2_ Nanofluids

**DOI:** 10.3390/nano10091796

**Published:** 2020-09-09

**Authors:** Muhammad Zia Ullah Khan, Emad Uddin, Bilal Akbar, Naveed Akram, Ali Ammar Naqvi, Muhammad Sajid, Zaib Ali, Md. Yamin Younis, Fausto Pedro García Márquez

**Affiliations:** 1 Department of Mechanical Engineering, School of Mechanical and Manufacturing Engineering (SMME), National University of Science and Technology, Islamabad 44000, Pakistan; engr.ziaullah1992@gmail.com (M.Z.U.K.); emaduddin@smme.nust.edu.pk (E.U.); ali_ammar@smme.edu.pk (A.A.N.); m.sajid@smme.nust.edu.pk (M.S.); zaib.ali@smme.nust.edu.pk (Z.A.); 2 Department of Mechanical Engineering, Mirpur University of Science and Technology (MUST), Mirpur-10250 (AJK), Pakistan; bilal.akbar@must.edu.pk (B.A.); myyounis.me@must.edu.pk (M.Y.Y.); 3 Department of Mechanical Engineering, Faculty of Mechanical Engineering, University of Malaya, Kuala Lumpur 50603, Malaysia; 4 Ingenium Research Group, University of Castilla-La Mancha, 13071 Ciudad Real, Spain

**Keywords:** laminar flow, conjugate heat transfer, dean vortices, Nusselt number, friction factor

## Abstract

A new micro heat exchanger was analyzed using numerical formulation of conjugate heat transfer for single-phase fluid flow across copper microchannels. The flow across bent channels harnesses asymmetric laminar flow and dean vortices phenomena for heat transfer enhancement. The single-channel analysis was performed to select the bent channel aspect ratio by varying width and height between 35–300 μm for Reynolds number and base temperature magnitude range of 100–1000 and 320–370 K, respectively. The bent channel results demonstrate dean vortices phenomenon at the bend for Reynolds number of 500 and above. Thermal performance factor analysis shows an increase of 18% in comparison to straight channels of 200 μm width and height. Alumina nanoparticles at 1% and 3% concentration enhance the Nusselt number by an average of 10.4% and 23.7%, respectively, whereas zirconia enhances Nusselt number by 16% and 33.9% for same concentrations. On the other hand, thermal performance factor analysis shows a significant increase in pressure drop at high Reynolds number with 3% particle concentration. Using zirconia for nanofluid, Nusselt number of the bent multi-channel model is improved by an average of 18% for a 3% particle concentration as compared to bent channel with deionized water.

## 1. Introduction

Intelligent systems being used in recent times come equipped with microprocessor chips which allow for increased functionality, occupy less space, and provide greater portability. While the development of microelectronic devices continues to provide convenience to humankind, however, the reduced durability of such devices due to their heat-sensitive nature remains a persistent problem.

In most practical applications, heat transfer between one or more fluids takes place through heat exchangers. For cooling of Micro-Electro-Mechanical Systems (MEMS), micro heat exchangers serve as a solution to the heating problem at small scales. With applications ranging from small phones to significant industrial heat generating units, the advantage of heat exchangers being used at micro-scale lies with their compact size, low material cost, mobility, and better performance. Much like their conventional counterparts, the micro heat exchanger consists of confined ducts that constrain the flow of fluid or gas stream. The higher surface area to volume ratio provided by microchannels results in reduced thermal resistance and higher heat transfer rates. The heat transfer rates for microchannel heat exchangers are dependent upon the performance of fluids, with the thermal conductivity of fluid restricting the achievable heat transfer rates. However, the introduction of nanoparticles inside the base fluid can help overcome such barriers. Such mixtures, known as nanofluids consist of a base fluid and solid particles, are developed by mixing a suspension of nanoparticles in base fluids.

In 1981, Tuckerman and Pease [1] demonstrated that a confined flow of liquid could aid in increasing convective heat dissipation from the electronic chip through the application of high aspect ratio microchannels. The detailed study by Peterson et al. [2] concluded that heat sinks perform at their best when the heat exchanger is properly in contact with the electronic device junction. Tuckerman [3] further performed numerical analysis on the efficient cooling system for dissipating 1000 W/cm^2^ heat while maintaining structure temperature inside the limit of the safe functionality of transistors. Moon and Jhong [4] numerically and experimentally investigated the cooling performance of micro heat exchanger on stacked multi-chip modules with single-phase laminar flow. Amirah et al. [5] performed numerical and experimental investigations for single and multi-channel micro heat exchanger configurations using a range of hydraulic diameters, concluding that transition from laminar to turbulent flow occurs at Reynolds number magnitude of 1600 and that the presence of significant conjugate effect results in the difference between numerical and experimental results. While working at a micro-scale, a question arises about whether or not the conventional flow and heat transfer theories can be safely applied to micro-scale flows. Some studies contradict conventional theories [6,7]. However, other studies were unable to find a significant difference between flow and heat transfer for conventional and micro-scale applications. The absence of difference concludes that discrepancies in literature can be a result of scaling effect and uncertainties which can arise due to viscous heating, surface roughness, properties dependent on temperature, and entrance and conjugate heat transfer [8,9,10,11]. While considering scaling effects, axial wall conduction is also a significant problem that contributes to the decrease in the thermal efficiency of the microchannel heat exchanger. Experimental and numerical studies demonstrate that, by increasing the thermal conductivity of the micro heat exchangers, axial wall conduction can also increase, resulting in lower overall thermal efficiency. Therefore, the conclusion is that low thermal conductivity materials are efficient for resisting axial wall conduction [12,13]. However, the use of low thermal conductivity material for heat transfer has limited applications.

Various studies utilize numerical simulations as a tool for predicting the behavior of microchannels, as they tend to provide flexibility in design aspects allowing for greater opportunity in exploring new channels and design variations. A numerical study on microchannel heat exchangers conducted by Liu and Garimella [14] showed that for laminar flow across channels both microscale and conventional channels have the same behavior for a range of hydraulic diameters. Xu et al. [15] numerically studied the flow characteristics for Reynolds number range of 20–4000, showing that flow characteristics in microchannels are similar to Navier–Strokes predicted behavior, suggesting that deviation in early studies could be due to error in dimensions. Hetsroni et al. [16] validated the available experimental data through numerical studies by considering hydrodynamic characteristics of laminar flow inside a micro heat exchanger with uniform flux while keeping necessary allowance for channel geometry, wall conduction, energy dissipation, and physical properties of the fluid. Allen et al. [17] experimentally and numerically investigated fluid flow and heat transfer in copper micro heat exchangers considering constant heat base temperature. Results from the experiments were found to be in good agreement with simulation results. Heat transfer enhancement in curved microchannel has been studied by many researchers concluding a significant increase in Nusselt number with little increase in pressure drop when the curved microchannel is compared to straight microchannel [18,19,20,21].

Sui et al. [22] performed experimental and numerical analysis on wavy microchannels, concluding that an increase in heat transfer was disproportionate to the increase in the corresponding increase in pressure drop for wavy microchannel due to the formation of dean vortices. Wang et al. [23] performed a numerical study on friction factor and Nusselt number in curved channels, concluding that the increase in mean Nusselt number and friction factor occurs as a result of vortex generation due to the effect of buoyancy.

Diverging-Converging plenum geometry of triangular-shaped was used due to its better flow distribution for the multi-channel model [4,24]. Sehgal et al. [25] studied convective heat transfer and pressure drop of the microchannel with top and side inlet plenum and found side inlet to be 13% more efficient. Balaji et al. [26] concluded inlet and outlet in-line with microchannel evenly distributes the pressure drop.

In recent times, the use of nano-scale technology has emerged widely and revolutionized several technological fields. Nanofluid technology is one specific area where a homogenous mixture of nanoparticles and a base fluid is used to develop nanofluids. Such fluids have vast applications in tribology [27,28], heat transfer enhancement [29,30,31,32], and biomedical filed [33,34,35].

Since these fluids form a colloidal solution of the solid-liquid particles, therefore, the computational domain for nanofluids can be evaluated using either Eulerian or Lagrangian approach, depending upon the application. For a simplifying model, to minimize the computational effort, in engineering applications, like heat exchanger, the Eulerian approach is preferred [29]. However, in biomedical application, the Lagrangian approach is considered more effective, as the primary focus is on the study of Brownian motions and interaction of individual particles [33,36].

Nanofluids are commonly used in heat transfer units for performance enhancement of cooling fluids. Convective heat transfer using nanofluids, like Al_2_O_3_ [37,38,39], ZrO_2_ [39,40], CNT [41,42], CuO [38], Cu [38], and TiO_2_ [43,44], have been investigated in numerical studies. Williams et al. [39] investigated the heat transfer and pressure drop in horizontal tubes under turbulent flow conditions by performing experimental analysis on setup using alumina-water (Al_2_O_3_)- and zirconia-water (ZrO_2_)-based nanofluids. Rea et al. [40] experimentally investigated convective heat transfer and pressure drop in vertical heated tubes in laminar flow conditions using Al_2_O_3_ and ZrO_2_ nanofluids with enhancement in heat transfer coefficient [45,46,47,48,49]. Thus, the design of geometry, selection of nanofluids types, and concentration contributes to obtaining high thermal effectiveness.

This study focused on the numerical analysis of bent microchannel for evaluating the behavior of the fluid flow and conjugate heat transfer. The microchannel design is the most critical parameter as it can be harnessed to achieve maximum heat transfer with a lesser corresponding increase in pressure drop. Geometry, flow, and temperature of the single microchannels are varied to achieve the desired performance. Variations in width, height, the hydraulic diameter of the channel for a range of Reynolds number, and base temperatures were studied. Analysis on a single channel with a 25-degree bend was performed by varying width and height to obtain the best geometric parameter for multi-channel. Alumina and zirconia nanoparticles were introduced in the optimum bended single channel to calculate Thermal Performance Factor (TPF). After the selection of a single channel, a multi-channel micro heat exchanger was analyzed with and without nanoparticles, providing a comprehensive design of micro heat exchanger with multiple design parameter considerations.

## 2. Materials and Methods

### 2.1. Geometric Configuration and Computational Domain

#### 2.1.1. Single Channel

**Figure 1:**
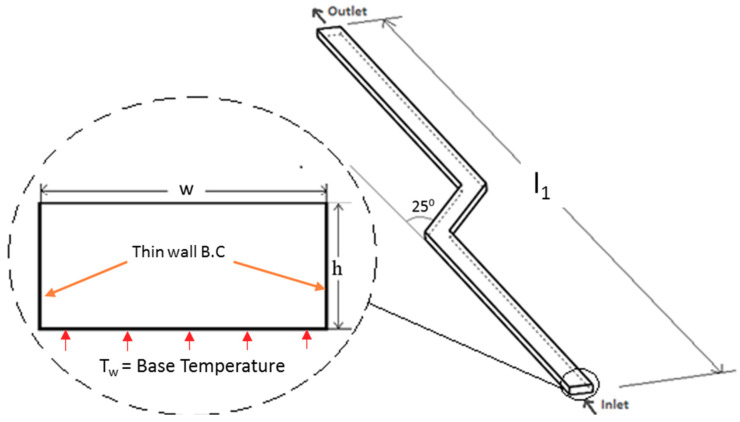
Single channel geometric parameters.

The numerical study of conjugate heat transfer problem accounts for convective heat transfer through fluid-solid interface and conductive heat transfer within both the solid and fluid domains. The geometry of single channel with fix length is shown in Figure 1. The effect of bend in the conventional straight channel was investigated to determine the changes in pressure drop and Nusselt number. The geometrical parameters of the channel with its width and height were varied between 35–300 μm. Table 1 provides dimensional details of single channel for optimum bend angle selected through numerical study. The length of a single channel was kept such that it covers the entire 28 × 7-mm cross-section of the electronic chip when introduced in the multi-channel model.

#### 2.1.2. Multi-Channel

**Figure 2:**
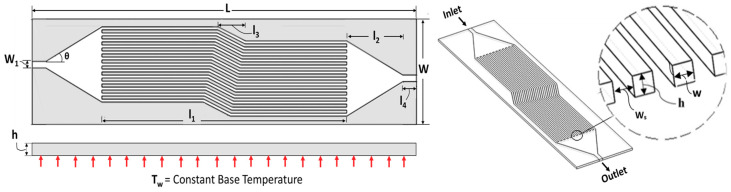
Multi-channel configuration (solid with grey color and fluid with white color).

The multi-channel configuration given in Figure 2 was developed using a series of single channels after carefully analyzing performance parameters for each geometric variation across single channels, as discussed in the previous section. The performance of multi-channel was investigated by varying channel height and base temperature at Reynolds number range from 100–1000 while keeping the length and width of all channels the same. The dimensional constraint for the multi-channel model is also given in Table 1.

### 2.2. Mathematical Formulation

The working fluid for the present case was de-ionized water with temperature-dependent properties, while the solid material was copper with fixed properties, as given in Table 2. The conjugate heat transfer problem was simplified using the below-mentioned assumptions and idealizations:

1. 3D incompressible fluid with the steady-state formulation.
2. Constant density and variable specific heat, viscosity, and thermal conductivity with respect to temperature is used for fluid. Viscosity, specific heat, and thermal conductivity are a piece-wise linear function of temperature, as shown by Peiyi et al. and Okhotin et al. [50,51].
3. Constant wall temperature is assumed, considering uniform temperature distribution throughout the channel base.
4. Constant solid properties are used, with side walls having the adiabatic condition.
5. Radiation and viscous dissipation are neglected.

By considering the above assumptions, governing equations for the description of fluid flow and heat transfer can be given as:

Conservation of mass:

(1) ∇.(ρV)=0;

Conservation of momentum:

(2) V.∇(ρV)=−∇p+∇.(μ∇V);

Conservation of energy (Fluid):

(3) $$V.\nabla \left({\rho C}_{p}T_{f}\right)=\nabla .\left(k_{f}\nabla T_{f}\right);$$

Conservation of energy (Solid):

(4) $$\nabla .\left(k_{\mathrm{so}}\nabla T_{\mathrm{so}}\right)=0.$$

The uniform velocity boundary condition was applied at inlet and pressure outlet condition at the outlet, as the flow was assumed to be incompressible. The sidewall boundaries were assigned no-slip condition, whereas top and sidewall were assigned adiabatic condition, and constant temperature was applied on the bottom wall. The boundary conditions given in Figure 1 show a thin wall boundary condition applied to the channel sides. It was assigned in ANSYS Fluent (Canonsburg, PA, USA) to model conjugate heat transfer without modeling solid geometry, and a fixed value of wall thickness for thin-wall model was assigned as half of the channel width.

### 2.3. Numerical Procedures and Parameter Definition

**Figure 3:**
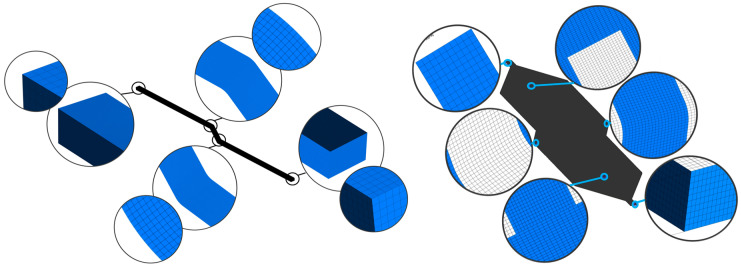
Single (Left) and Mmulti-channel (Right) mesh.

The design, meshing, and analysis tools available within ANSYS 18.1 (Canonsburg, PA, USA) were used for the study of microchannels. The design modeler tool was used to develop the model, and the inbuilt meshing tool was used to generate a structured non-uniform mesh using hexahedron elements, as shown in Figure 3, for computational domain discretization. The mesh was accomplished in the meshing module with minimum mesh orthogonality of 0.96734, the maximum aspect ratio of 1.2, and a maximum skewness of 0.16. The viscous laminar model was used with a Semi-Implicit Method for Pressure Linked Equations (SIMPLE) scheme for solving pressure velocity coupling [5]. Second-order upwind scheme was used for solving momentum and energy equations. Converging criteria for governing equations were set to 10^−5^. The average mesh elements for single-channel were 3.2 million, whereas, for the multi-channel model, the average mesh elements were 25 million. The analysis was performed using the Fluent module within ANSYS 18.1 software, and computation was performed on dual Xeon X5650 12 core processor with 72 gigabytes RAM. The average computation time for a single channel model was two hours, whereas the average computation time for the multi-channel model was 48 h.

#### 2.3.1. Friction Factor

The numerical results are evaluated in terms of certain performance parameters, namely friction factor and Nusselt number. The Reynolds number (Re) is a function of liquid density (ρ); dynamic viscosity (μ); and Inlet velocity $v_{in}$ of fluid, and hydraulic diameter $\left(D_{H}\right)$ of the channel is defined by Equation (5):

(5) Re=ρDHvinμ,

where

(6) $$D_{H}=\frac{2\mathrm{wh}}{\left(w+h\right)}.$$

The friction factor and pumping power is calculated by Equations (7) and (8):

(7) $$f=\frac{D_{H}}{L_{c}}\frac{2\;\Delta P}{{\rho \;v}_{\mathrm{in}}^{2}},$$

(8) $$P_{\mathrm{power}}=\Delta P\;\dot{V},$$

where: $\dot{V}$ is the volume flow rate of fluid, $L_{c}$ is the length of the microchannel understudy, and ΔP is the pressure difference calculated by subtracting pressure of outlet going fluid from inlet coming.

Hansel [53] suggested a friction factor equation for the analytical formulation of pressure drop by substituting f in Equation (9).

(9) f=64Re[23+11h24w(2−hw)].

Upadhye et al. [54] used pressure drop and Poiseuille number equation to analytically find pressure drop in a microchannel.

(10) ΔP=2Poρ vin2ReLDH.

Poiseuille number equation in friction factor form is expressed by Equation (11) [55]:

(11) $$P_{o}=\mathrm{fRe}.$$

In Equation (11), the value of $P_{o}$ can be calculated from Shah and London [56] for the microchannel:

(12) $$P_{o}=24\left(1-1.13553\alpha_{\mathrm{ch}}+1.9467\alpha_{\mathrm{ch}}^{2}-1.7012\alpha_{\mathrm{ch}}^{3}+0.9564\alpha_{\mathrm{ch}}^{4}-0.2537\alpha_{c}^{5}\right),$$

where $\alpha_{\mathrm{ch}}$ is the aspect ratio of the microchannel.

#### 2.3.2. Heat Transfer

Heat transfer co-efficient values h is necessary to determine microchannel thermal performance. Non-dimensional parameter Nusselt number is considered for heat transfer evaluation of micro heat exchanger. Nusselt number (Nu) is expressed by Equation (13):

(13) $$\mathrm{Nu}=\frac{{\mathrm{hD}}_{H}}{k}.$$

h can also be represented in total heat rate form by Equation (14):

(14) $$h=\frac{Q}{A_{\mathrm{ht}}\;\Delta T},$$

where ΔT is the difference in temperature between the solid surface and flowing fluid, and

(15) $$Q={\dot{m}C}_{p}\left(T_{o}-T_{i}\right).$$

The energy balance depicting fluid flow through a microchannel, with assumptions of constant base temperature (T_w_), negligible accumulation of mass, and no change in property with time, is given by Equation (16):

(16) $$C_{p}\dot{m}\left(T_{o}-T_{i}\right)={\mathrm{hA}}_{\mathrm{ht}}\frac{T_{o}-T_{i}}{\ln\left(\frac{T_{w}-T_{i}}{T_{w}-T_{o}}\right)}.$$

Equation (15) can be substituted into Equation (16) for obtaining non-dimensional Nusselt number (Nu) [57] form given by Equation (17):

(17) $$\mathrm{Nu}=\left(\frac{D_{H}}{k}\right)\ln\left(\frac{\left(T_{s}-T_{i}\right)}{\left(T_{s}-T_{o}\right)}\right)\left(\frac{{\dot{m}C}_{p}}{A_{\mathrm{ht}}}\right),$$

where k is the thermal conductivity of fluid at mean temperature; $C_{p}$ is the specific heat of fluid at mean temperature; $T_{s}$ is bottom wall temperature; $T_{i}$ is fluid inlet temperature; $\dot{m}$ is the mass flow rate of fluid; $T_{o}$ fluid outlet temperature; and $A_{\mathrm{ht}}$ is the area of base at which temperature is applied.

#### 2.3.3. Nanofluids

The heat transfer by nanofluids depends on their thermos-physical properties that are a function of nanoparticle volume percentage (∅) in relation to the properties of water and nanoparticles [39,40]. The density and specific heat of nanoparticles is defined as follows:

(18) $$\;\emptyset {\;\rho }_{\mathrm{np}}+\left(1-\emptyset \right)\;\emptyset \rho_{f},$$

(19) $$\frac{\;\emptyset {\;\rho }_{\mathrm{np}}C_{\mathrm{np}}+\left(1-\emptyset \right){\;\rho }_{f}C_{f}}{\rho_{\mathrm{nf}}},$$

where ${\;\rho }_{\mathrm{np}}$ and ${\;C}_{\mathrm{np}}$ represent density and specific heat of nanoparticles. The National Institute of Standards and Technology (NIST) database was used for alumina and zirconia properties (Table 3).

The temperature-dependent thermal conductivity ($k_{\mathrm{nf}})$ and viscosity $\left(\mu_{\mathrm{nf}}\right)$ of nanofluids in curve fitting form is expressed as follows:

Alumina-water:

(20) $$k\left(\emptyset ,T\right)=k_{f}\left(T\right)\left(1+4.5503\emptyset \right),$$

(21) $$\mu \left(\emptyset ,T\right)=\mu_{f}\left(T\right)\;\exp\left[\frac{4.91\emptyset }{\left(0.2092-\emptyset \right)}\right].$$

Zirconia-water:

(22) $$k\left(\emptyset ,T\right)=k_{f}\left(T\right)\left(1+2.4505\emptyset -29.867\emptyset^{2}\right),$$

(23) $$\mu \left(\emptyset ,T\right)=\mu_{f}\left(T\right)\left(1+46.801\emptyset +550.82\emptyset^{2}\right).$$

The constraint of the temperature range for these equations is between 20 °C and 80 °C for the volumetric concentration of 6% in the case of alumina and 3% for zirconia.

Equation (24) represents TPF, which is utilized as a parameter for the selection of microchannel by comparing the thermo-hydraulic performance of channels.

(24) $$\mathrm{TPF}=\frac{\frac{\mathrm{Nu}}{{\mathrm{Nu}}_{o}}}{{\left(\frac{f}{f_{o}}\right)}^{\frac{1}{3}}}.$$

## 3. Results and Discussion

### 3.1. Grid Independence

**Figure 4:**
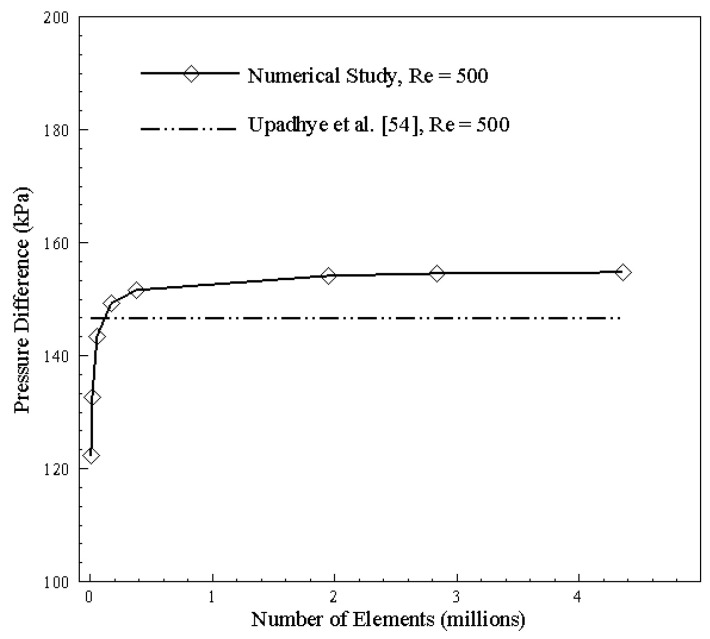
Grid independence and comparison with the analytical solution.

Since the finite volume method involves discretization of the domain into a finite number of volumes, grid independence is the first necessary step towards the numerical solution of problems. Grid independence studies are conducted on successively dense mesh sizes to predict a stage where the result stabilizes and the variation between numerical and analytically formulated results becomes almost negligible. The grid independence in terms of pressure drop magnitudes has been shown both in graphical and tabular form in Figure 4 and Table 4, respectively. A closer look at the pressure drop curve shows that, for a coarse mesh, the simulation error is significant; however, as the mesh becomes relatively dense, the numerical solver can capture additional pressure losses across the bends and provides a much more accurate solution as we approach 1 million mesh elements. Upon further increasing the mesh density, we observe stabilization of results as the number of elements is increased from 2.8 to 4.3 million with no discernable increase in pressure drop magnitude. Therefore, a mesh of 4.3 million elements having an acceptable error of 5.5%, in comparison with the analytical solution given by Upadhye et al. [54]’s relation, is chosen as the optimum size.

### 3.2. Data Validation and Reduction

**Figure 5:**
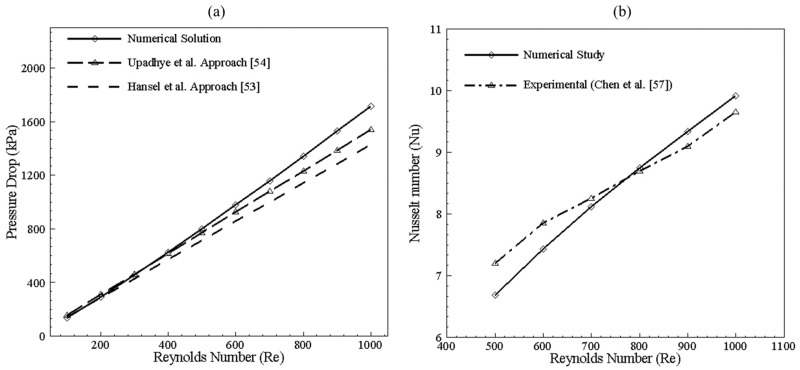
(**a**) Pressure drop validation for microchannel. (**b**) Nusselt number validation for microchannel.

The Poiseuille number approach used by Upadhye et al. [54] was used to validate the pressure drop in bended channels. The results were also counter-validated using the Hansel relation [53]. Both these relations provide analytical results for straight channel configuration, and the graph given in Figure 5 shows the numerical data validation for a single bend channel. It can be seen that, at a low Reynolds number, straight channel equations suit well with a 25-degree bend channel, but, at high Reynolds number values, the effect of bend becomes prominent, and error in pressure drop increases to a maximum of 8% for Upadhye’s and 12% for Hansel’s solution. At a low Reynolds number, the effect of dean vortices is not prominent, resulting in no increased pressure drop; therefore, the results are in good agreement with straight channel relations. However, an increase in Reynolds number enhances the formation of dean vortices. Hence, the effect of bend becomes prominent. Thus, numerical simulation defies Upadhye’s and Hansel’s approach at a high Reynolds number. The Nusselt number was validated through the experimental study of Chen et al. [57], showing a maximum of 8% error when compared with numerical study for Reynolds number range of 500 to 1000.

**Figure 6:**
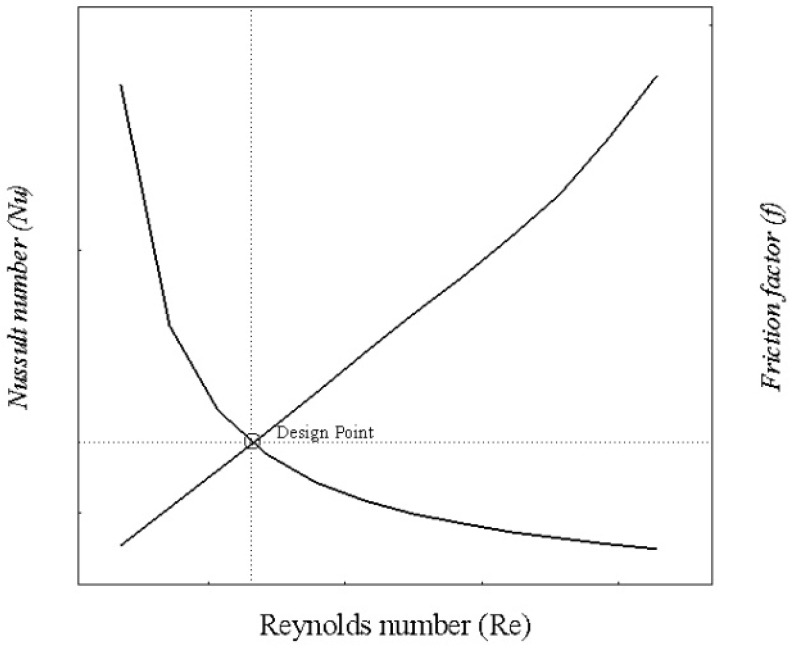
Design point selection depending upon performance parameters.

Design of microchannel involves a trade-off between Nusselt number and friction factor since they represent the convective heat transfer rate and pressure drop, respectively. For single-channel models, a total of 360 cases are formulated for a Reynolds number range of 100–1000 at varying cross-sections and base temperatures. For data reduction, a total number of 360 single-channel case studies are reduced to 64 by using the design point approach. The design point approach involves the selection of optimum points by plotting the Nusselt number (Nu) and friction factor (f) at different Reynolds numbers. The intersection points of these curves are chosen as the design point for each specific model, as shown in Figure 6.

### 3.3. Bend Selection

**Figure 7:**
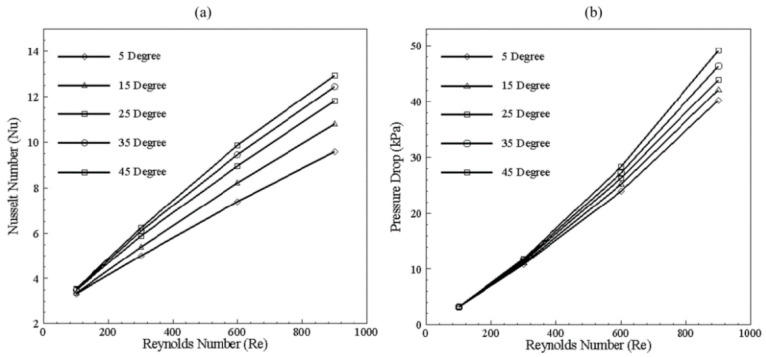
(**a**) Nusselt number variation with change in bend angle at different Reynolds numbers. (**b**) Pressure drop variation with change in bend angle at different Reynolds numbers.

**Figure 8:**
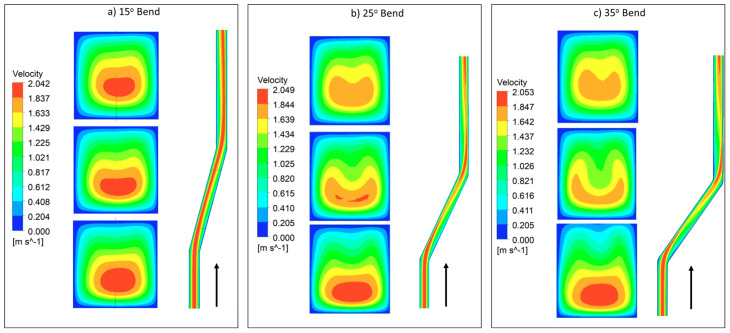
Velocity contours formation at different bend angles for Re = 300. (**a**) Contours at different cross-sections of 15° bend. (**b**) Contours at different cross-sections of 25° bend. (**c**) Contours at different cross-sections of 35° bend.

The selection of bend is executed by considering geometric constraints as chip size and the number of channel adjustments in the multi-channel model. Furthermore, performance evaluation in the form of Nusselt number and pressure drop, as well as the formation of dean vortices, is also considered. By considering geometric constraints angle is limited to range 0° to 45°. Figure 7 depicts graphical information about Nusselt number and pressure drop for 200 × 200 μm channel at different Reynolds numbers. It can be observed from Figure 7a that, until 300 Reynolds number, no significant change in Nusselt number is visible by changing the bend angle. However, at 300 and above, improvement is significant, which can be correlated with velocity contours in Figure 8. Furthermore, with increment in the Reynolds number, a rise in pressure drop can be seen in Figure 7b.

It can be observed that, after 15° change in angle, a sudden increase in Nusselt number with Reynolds number is visible compared to change in angle from 25° to onwards. A comparison of Figure 7a and Figure 8 demonstrates that, due to the generation of dean vortices in the 25° bend channel, Nusselt number increased. It is identified that, with the 25° to onward bend, Nusselt number and pressure drop increases in equal proportion. Therefore, considering the increase in Nusselt number, generation of dean vortices, and consideration of geometric constraints, 25° angle was selected for the multi-channel model. The longitudinal cross-section of the microchannel in Figure 8 depicts the mixing of fluid by variation in its contours at the bend region.

The study focuses on improving convective heat transfer by introducing a bend in the straight channel, which will increase convective heat transfer by asymmetric laminar flow, dean vortices generation, and increased flow length of the channel. Since this configuration provides the dean vortices and increased fluid contact with a heated surface, the pressure drops and heat transfer magnitudes for such bended channels are studied. From gathered design points, the 25° angle is selected, and a comparison with the straight channel of the same length is made, which shows a 5.3% increase in Nusselt number, while an increase in pressure drop is 3.3%, as shown in Figure 5. The angle is selected while considering the Nusselt number and pressure drop as performance parameters and space covered as a geometric parameter. The occupied space due to bend is of most concern as an increase in angle will widen the overall size of a single channel, creating difficulty in the multi-channel arrangement of bend channels.

**Figure 9:**
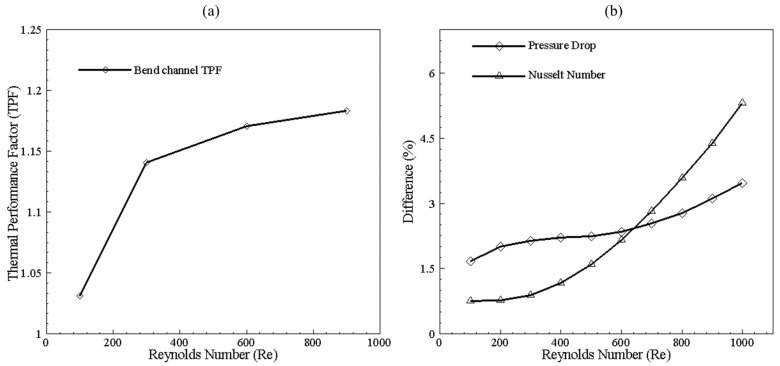
(**a**) Thermal Performance Factor (TPF) of bend channel (200 by 200 μm) with the straight channel (200 by 200 μm) as a benchmark. (**b**) The performance difference of bend channel (200 by 200 μm) with the straight channel (200 by 200 μm).

**Figure 10:**
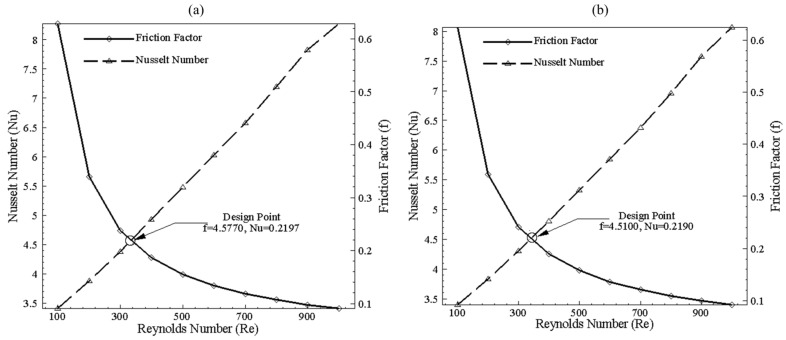
Nusselt number (Nu) and Friction factor (f) for 200 μm width and 100 μm height at (**a**) 320 K and (**b**) 325 K temperature.

The performance of 200 × 200 μm in the form of thermal effects can be seen in Figure 9a, depicting TPF greater than 1 for Reynolds number from 100–900. TPF value greater than 1 represents more heat transfer than pressure drop when bend channel is compared with the benchmark of the straight channel. Thus, bend will enhance thermal performance. Figure 9b illustrates that using the bend channel improves Nusselt number with increasing Reynolds number when compared with the straight channel of the same dimension of 200 × 200 μm; however, increment in pressure drop can also be observed. Thus, for the single channel case, a significant effect of Nusselt number can be achieved when the Reynolds number is higher. Figure 10 depicts the selection of the design point for 200 × 100 μm width and height channel at 320 K and 325 K temperature. With the increase of Reynolds number, lower values of friction factor can be observed, whereas the overall Nusselt number increases. It can be seen that, by varying base temperature, points of intersection of Nusselt number and friction factor changes, showing a trend that will be discussed for all single channel configurations. Data is reduced by plotting only design points in further study for all combinations of cross-sectional dimensions.

### 3.4. Single Channel Configuration

The 25° bend angle is employed in the study of microchannel at different cross-sections to find the optimum aspect ratio for multi-channel consideration. Table 5 shows a range of width and height for optimum selection.

Following the same technique of design point selection optimum points of pressure drop and Nusselt number for G1, G2, G3, and G4 were gathered. The surface contour of the design point values of friction factor and Nusselt number is generated and discussed.

**Figure 11:**
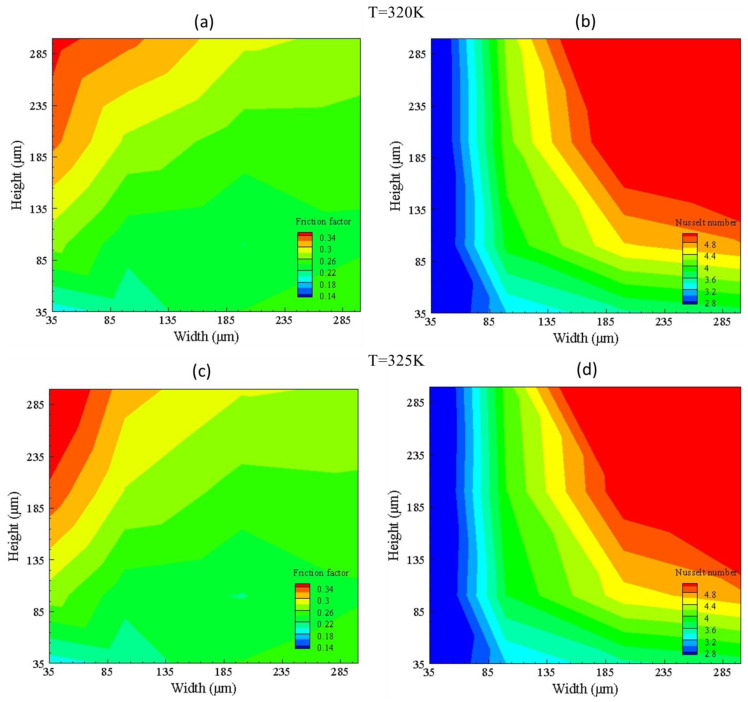
Friction factor (**a**,**c**) and Nusselt number (**b**,**d**) contours for group G1 (320 K) and G2 (325 K).

The friction factor and Nusselt number for G1 and G2 at 320 K and 325 K can be seen in Figure 11. It depicts that friction factor increases slightly when the width is varied by keeping height between 35 to 100 μm; however, high friction factor is obtained by varying height, while keeping width between 35 to 100 μm. By keeping a width between 35 to 100 μm and varying height minimum, the Nusselt number value is obtained due to less area in the bottom surface; thus, convective heat transfer is less. However, maximum Nusselt number is obtained above the width and height of 150 μm due to sufficient convective heat transfer; hence, a threshold limit for width and height is found.

**Figure 12:**
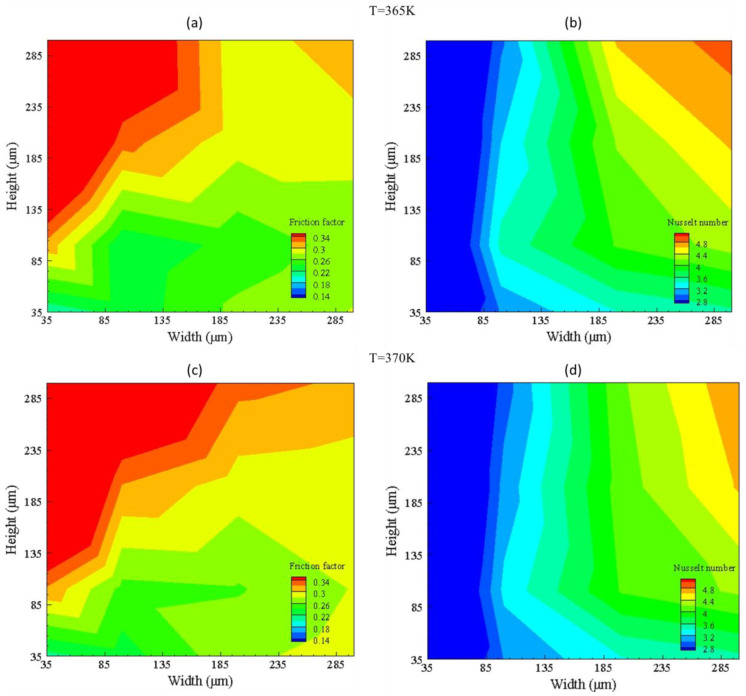
Friction factor (**a**,**c**) and Nusselt number (**b**,**d**) contours for group G3 (365 K) and G4 (370 K).

The high temperature analysis represented in Figure 12 shows the same behavior as that of friction factor and Nusselt number in the case of low temperatures; but, for high temperature, high maximum friction factor and low Nusselt number is seen due to the increase of friction at high temperature as the fluid excitation energy increases on heating and thus increases resistance. For the Nusselt number, at high temperature, convective heat transfer is reduced, resulting in increased conductive heath transfer hindering fluid capability of carrying heat. Nusselt number can be increased by increasing the flow rate, but this will compromise the friction factor, resulting in high pressure drop.

**Figure 13:**
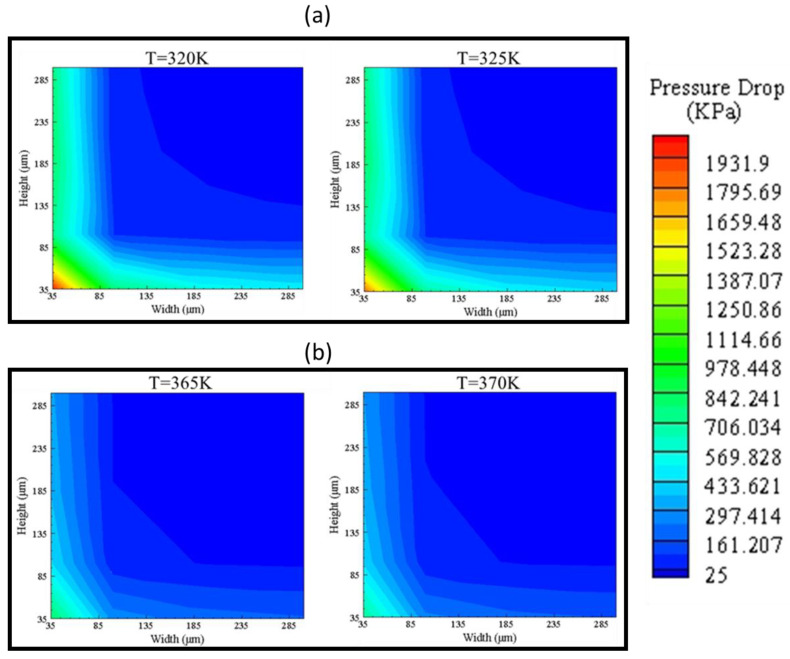
Pressure drop contours of (**a**) G1, G2 and (**b**) G3, G4.

The pressure drops across the microchannel is an essential factor in designing the micro heat exchanger as it contributes to the selection of a pump. Figure 13 shows pressure drop contours of design points showing that, for low temperature, the width of 35 μm and variation of height results in high pressure drop, whereas, in its inverse case, pressure drop is less, which is due to increased contact area by increasing the width, reducing fluid viscosity, and resulting in less pressure drop. For high temperatures, the same effect can be seen. Visualizing pressure drop from low temperature (320 K) to high temperature (370 K) shows that pressure drop decreases due to a decrease in viscosity by an increase in temperature.

**Figure 14:**
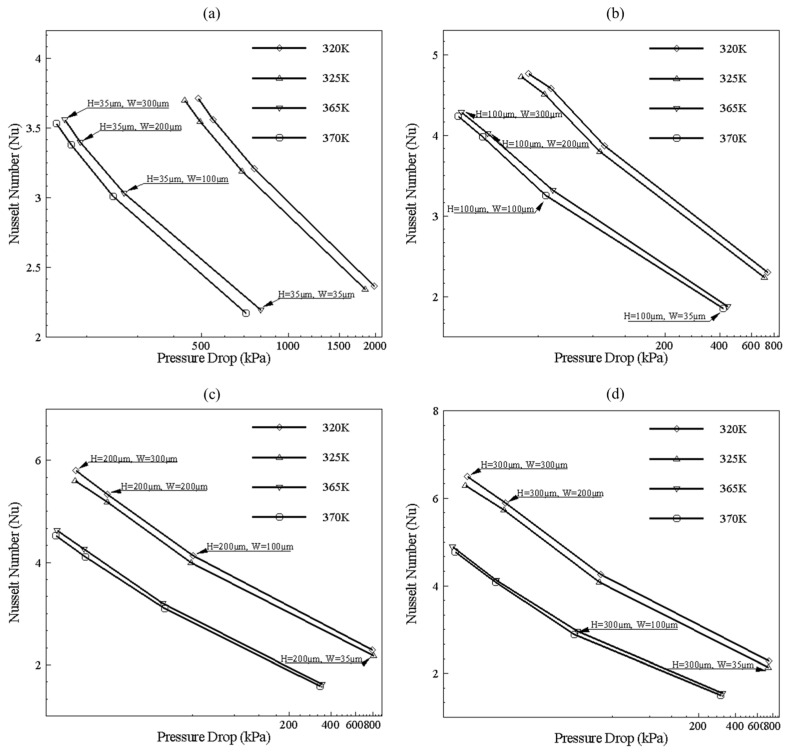
Channel selection based on pressure drop and Nusselt number (Nu). (**a**) Channel data with height 35 μm and width varying from 35–300 μm, from right to left in graph points, (**b**) Channel data with height 100 μm and width varying from 35–300 μm, from right to left in graph points, (**c**) Channel data with height 200 μm and width varying from 35–300 μm, from right to left in graph points, and (**d**) Channel data with height 300 μm and width varying from 35–300 μm, from right to left in graph points.

Results of pressure drop and Nusselt number of all geometric ranges for both width and height from 35 to 300 μm is shown in Figure 14. For low temperature, maximum Nusselt number and minimum pressure drop can be achieved with a height of 200 μm and a width of 300 μm. Likewise, for high temperature, the same width and height show the best performance, but pressure drop and Nusselt number values of 365 K and 370 K is lower than 320 K and 325 K. The trend is shown in Figure 14 is different from typical behavior like in Figure 10 because it involves plotting of intersection design points at different width and height values. It can be observed that, as base temperature is increased, pressure drop decreases with little decrement in Nusselt number, as compared to the geometry of the same parameter. The reason for the decrement of pressure drop is due to a decrease in fluid viscosity and Nusselt number decrease due to an increase in thermal boundary layer thickness, which decreases temperature gradient.

**Figure 15:**
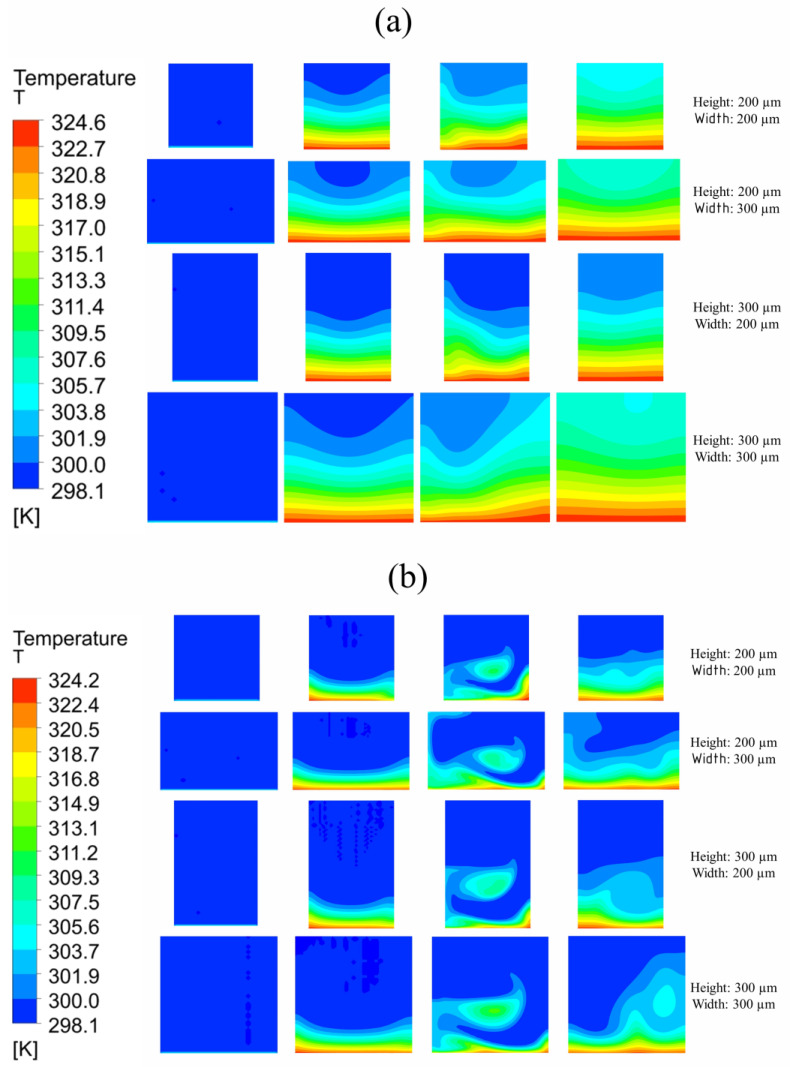
Temperature distribution in single microchannel at different cross-sections with 325 K base temperature: (**a**) Reynolds number = 100; (**b**) Reynolds number = 900.

Figure 15 demonstrates cross-sectional planes of the microchannel selected by the design point method. Temperature contours of the channel with 325 K base temperature can be seen at the inlet (x = 0), before bend (x = 8 mm), after bend (x = 20 mm), and at the outlet (x = 18). It can be seen that, for Reynolds number 100 and 900, 200 × 300 μm width and height channel are unable to carry a sufficient amount of heat from inlet to outlet, whereas the other three channels are very useful. Furthermore, the purpose of a bend can be seen clearly for Reynolds number 900 wherein heat is diffusing more in the channel as fluid moves across channel bends.

**Figure 16:**
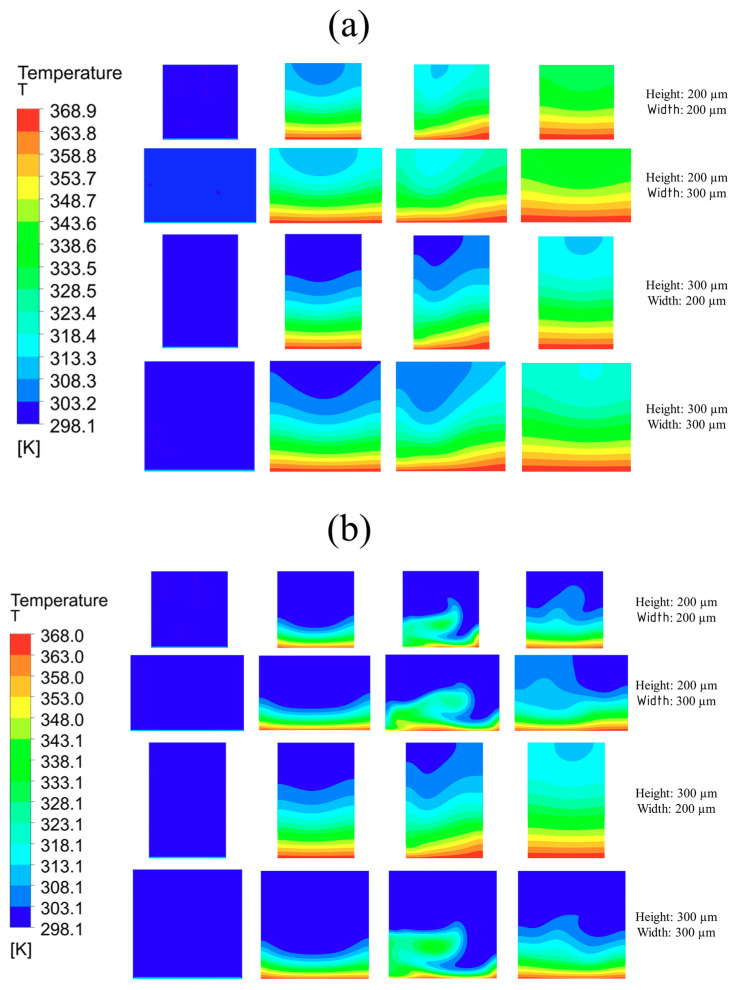
Temperature distribution in single microchannel at different cross-sections with 370 K base temperature: (**a**) Reynolds number = 100; (**b**) Reynolds number = 900.

Temperature contours in Figure 16 show that, for Reynolds number 100, channels with 300 × 200 μm width and height, respectively (rectangular), as well as 200 × 200 μm (square) channel, performed best in dissipating heat from inlet to outlet. However, for Reynolds number of 900, all channels other than of 200 × 300 μm width and height, respectively, showed good performance due to high base contact area.

#### Flow Behavior at the Bend

**Figure 17:**
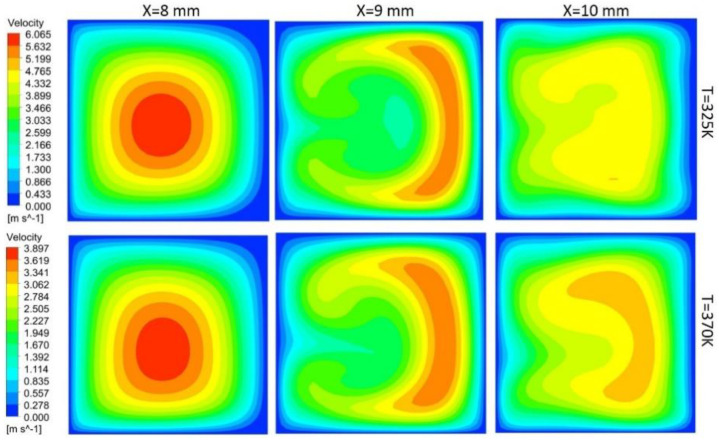
Dean vortices at 200 μm width and 200 μm height for Re = 600.

**Figure 18:**
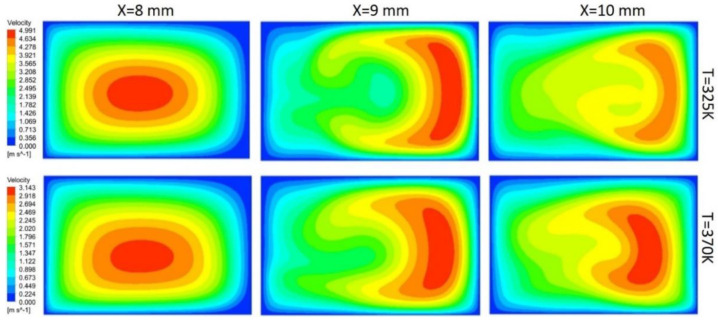
Dean vortices at 300 μm width and 200 μm height for Re = 600.

**Figure 19:**
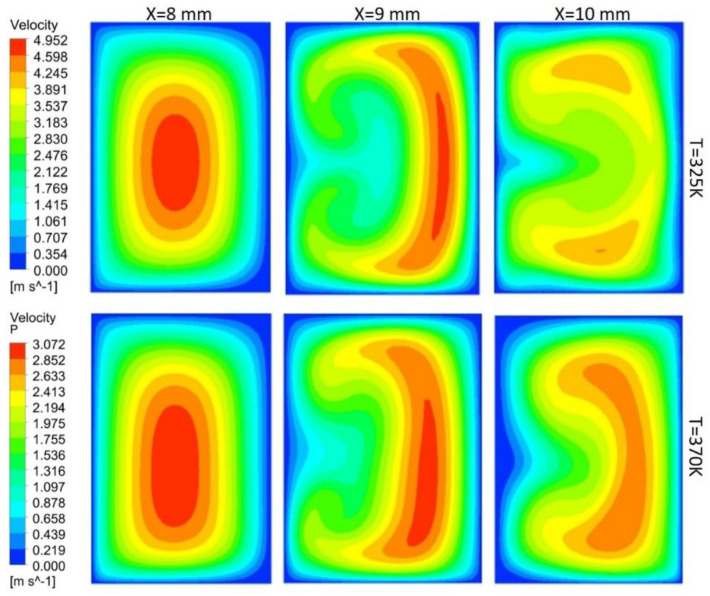
Dean vortices at 200 μm width and 300 μm height for Re = 600.

**Figure 20:**
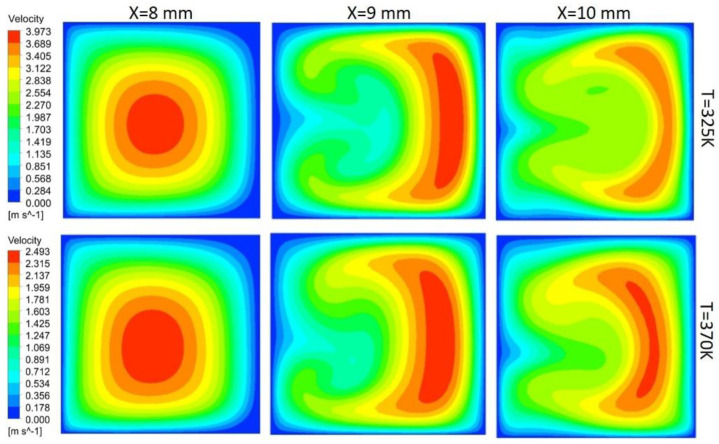
Dean vortices at 300 μm width and 300 μm height for Re = 600.

In this study, flow behavior at the bend involves the generation of secondary or dean vortices after Reynolds number value of 500. Velocity contours are shown in Figure 17 for 200 μm width, and height depicts that, at the initial edge of bend (x = 8 mm), no vortices are available, but the effect of the bend is starting to appear on outer edges, whereas at the middle of bend, counter-rotating vortices are visible because of velocity difference phenomena. Vortices help in the diffusion of heat and increase convective heat transfer at the expense of pressure drop in the channel. At the outer edge of the bend, the vortices effect becomes minimum. Non-symmetric behavior of the velocity contours is due to an increase in the kinetic energy of fluid molecule, which increases the average speed of molecules, and, as the channel is heated only from bottom side, behavior of velocity contours is developed accordingly. Figure 17, Figure 18, Figure 19 and Figure 20 depict velocity behavior at a low base temperature of 325 K and at a high base temperature of 370 K, where velocity difference can be observed due to temperature dependent fluid parameters.

Figure 18 depicts the behavior of dean vortices in the channel of 300 μm width and 200 μm height. The velocity of a fluid on the outer wall is more whereas the effect of vortices at the inner wall is less due to more width of the channel than the height, making less convective heat transfer. Vortices in Figure 19 are more symmetric than others due to less contact of channel base with fluid; hence, less fluid particles kinetic energy can be observed through contours. Comparison of the channel with 300 μm width and 200 μm height (rectangular shape) with the channel of width and height 300 μm (square channel) vortices from Figure 18 and Figure 20 shows that more heat diffusion is possible in the square channel due to more vortices’ dispersion in the square channel.

Kang et al. [4] study showed that an increasing number of channels increases the performance of a micro heat exchanger. In addition, flow uniformity increases by using microchannel with a small width, long length and sizeable manifold area [26,58]. Hasan et al. [59] studied the effect of channel geometry on micro-heat exchanger performance and concluded that the square channel provided the best overall performance than a rectangular channel.

**Figure 21:**
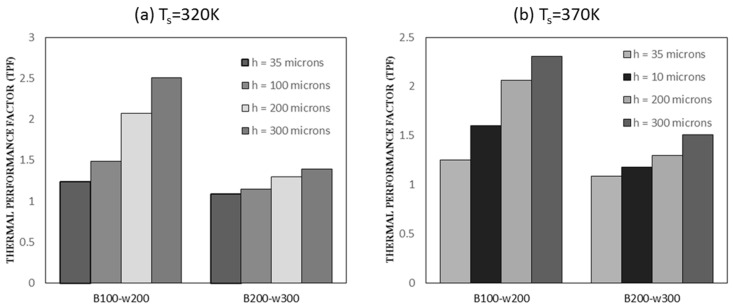
(**a**) TPF for single channel with 320 K base temperature. (**b**) TPF for single channel with 370 K base temperature.

TPF was used to evaluate the overall thermos-hydraulic performance of the channel by comparing the performance of the required channel with the benchmark channel. Figure 21 depicts TPF found using Equation (24), where Nu and f represent Nusselt number and friction factor of required channel, and $Nu_{0}$ and $f_{0}$ represent that of benchmark channel. TPF of more than one represents a higher convective heat transfer than the benchmark channel. In Figure 21, B100 represents a benchmark width of 100 microns, and w200 represents a width of 200 microns for the channel in which performance is to be compared. It can be observed clearly that the transfer of channel width from 100 microns to 200 microns shows greater TPF value as compared to width change from 200 microns to 300 microns. The width of 200 microns is satisfied for a low base temperature of 320 K to a high base temperature of 370 K.

**Figure 22:**
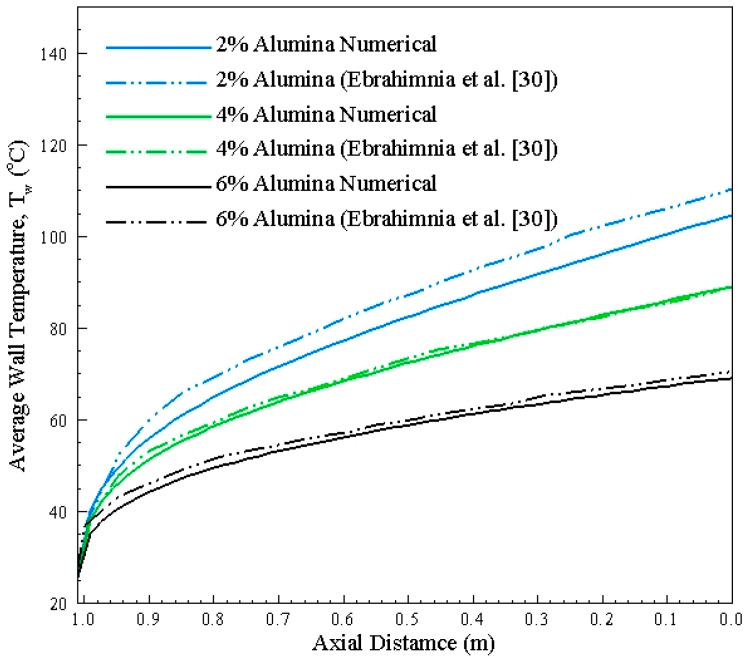
Average wall temperature (T_w_) along the length of channel.

Figure 22 portrays the validation of single microchannel considering different concentrations of alumina nanoparticles. The numerical methodology predicts the performance of channel with at maximum of 8% difference from reference study [30]. It can be observed that increase in concentration reduces the wall temperature, thus improving the cooling effect.

**Figure 23:**
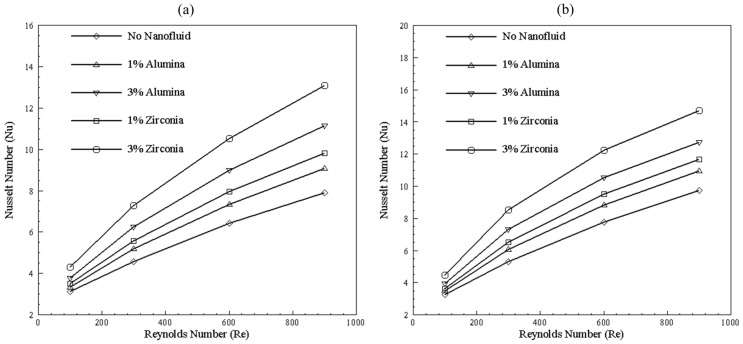
Effect of nanoparticles type and concentration on (**a**) straight and (**b**) bend channel.

Figure 23 clearly depicts that, for Reynolds number value of 100, no significant difference in Nusselt number is available, whereas, for higher Reynolds number, a surge in Nusselt number in the case of the bend channel is prominent. It can be observed that the overall trend of Nusselt number remains same with increase in Reynolds number for alumina and zirconia, with and without concentration. Furthermore, zirconia showed a higher Nusselt number than alumina for 1% and 3% of nanoparticles concentration. Curve fitting correlation for Nusselt number variation with Reynolds number range from 100 to 1000 Reynolds number is represented in Table 6. The average R-squared value of these equations is 0.99.

**Figure 24:**
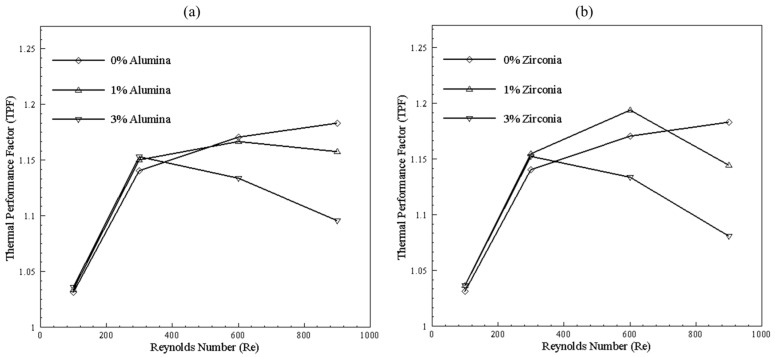
TPF of 200 μm width and height bend channel with (**a**) alumina concentration and (**b**) zirconia concentration with straight channel as benchmark.

TPF represents effectiveness of heat transfer enhancement technique. Figure 24 depicts effectiveness of bend channel by considering straight channel of same nanoparticles concentration as benchmark. It can be seen that using nanofluids enhances the heat transfer, as TPF in all cases is above 1. It can be observed that, for higher Reynolds number, friction factor dominates; thus, the slope of TPF starts decreasing at high Reynolds number. Furthermore, for 3% nanoparticles concentration, for both nanoparticles, the lowest performance is observed, depicting increasing in friction factor. However, when best performance is considered in comparison to without nanoparticles, 1% alumina concentration at Reynolds number of 300 standout. Likewise, for zirconia 1%, concentration with 600 Reynolds number proved best.

### 3.5. Multi-Channel Configuration

#### 3.5.1. Multi-Channel without Nanoparticles

Thus, the width of 200 microns is adjusted for multi-channel micro heat exchanger analysis. The number of channels is set to 13 for adjusting 200-micron channel in the complete multi-channel chip as depicted in Figure 2. Table 7 provides information about cases for multi-channel.

**Figure 25:**
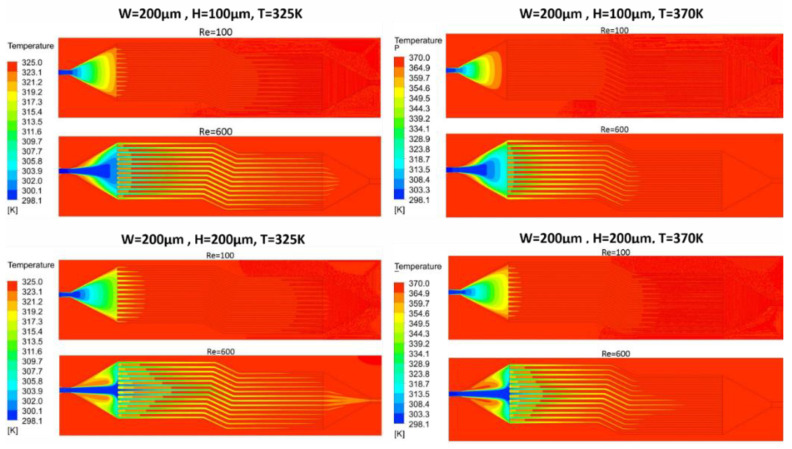
Temperature distribution in multi-channel for 325 K and 370 K temperature.

**Figure 26:**
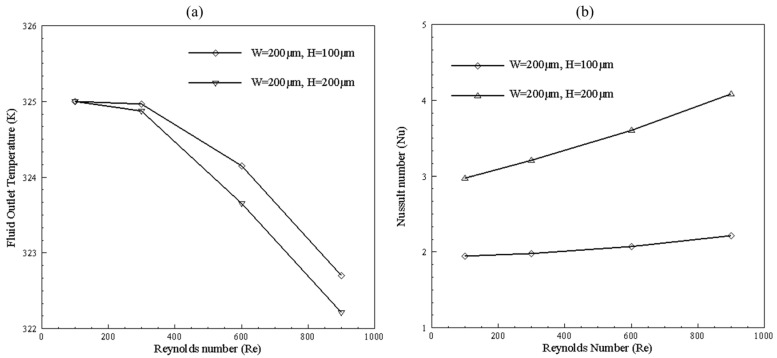
(**a**) Fluid outlet temperature and (**b**) Nusselt number at different Reynolds number.

Figure 25 shows the temperature distribution of two multi-channel models for Reynolds number 100 and 600. The outlet temperature of the channel with the width and height of 200 × 100 μm is higher than a channel with width and height of 200 × 200 μm for both high and low temperatures, but Nusselt number of the channel with 200 × 200 μm width and height is more significant due to more mass flow rate and bigger hydraulic diameter, as can be seen in Figure 26. Furthermore, fluid reaches near base temperature earlier in 200 × 100 μm channel. It can be seen that the temperature in the middle of microchannel reaches the base temperature slower than side walls due to the higher velocity in the middle of the microchannel.

Some high temperature zones can be seen after inlet for Reynolds number of 600. Low temperature area is due to the high velocity of fluid having less time to carry heat, whereas high temperature fluid contour is due to the low velocity of the fluid at expansion after entering plenum. By varying height from 100 to 200 μm, increase in Nusselt number is observed from 33% to 50%.

#### 3.5.2. Multi-Channel with Nanoparticles

**Figure 27:**
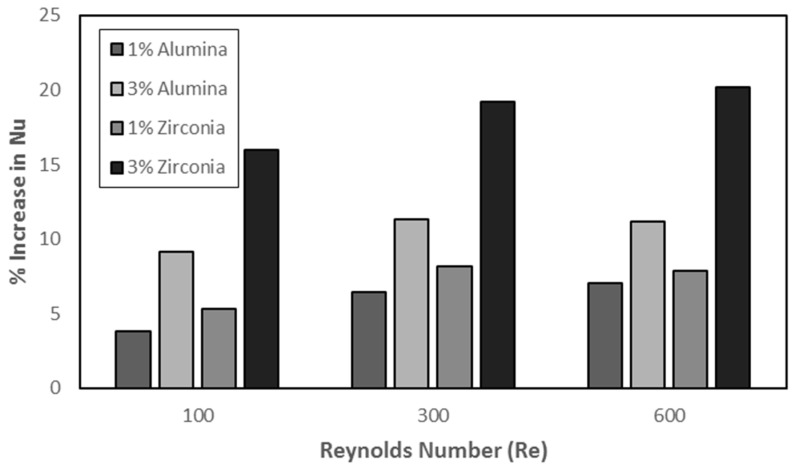
Percent increase in Nusselt number (Nu) by adding nanoparticles in multi-channel.

The effect of nanoparticles on Nusselt number (Nu) can be observed in Figure 27 in the form of percentage increase. The Nusselt number in case of zirconia is more than alumina showing a maximum of 20% increase in convective heat transfer at Reynolds number of 600. Furthermore, increase in concentration of particles enhanced the heat transfer. For concentration increase, a surge in performance can be observed for zirconia with concentration shift from 1% to 3%.

**Figure 28:**
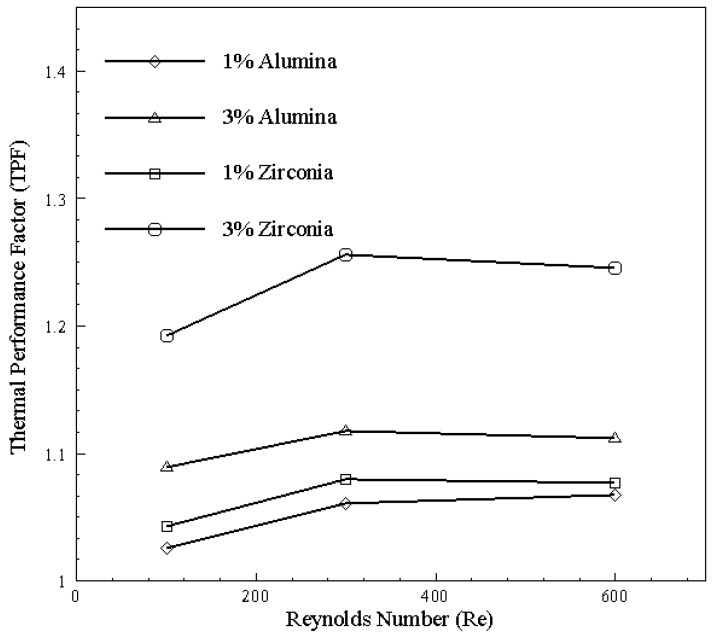
TPF of multi-channel micro heat exchanger with bend channel as benchmark.

The effectiveness of multi-channel micro heat exchanger given in Figure 28 with respect to bended channel as base model depicts that zirconia with 3% particle concentration is suitable for heat transfer by exhibiting increased cooling in comparison to increased viscous losses due to nanoparticles. Moreover, with increase in Reynolds number, pressure drop in the channel with nanoparticles starts dominating, resulting in decrement of TPF slope.

Table 8 represents the Nusselt number correlations for 100 to 600 Reynolds number range with R-square value of 1.

## 4. Conclusions

Numerical study was performed on bended microchannels to investigate heat transfer and fluid flow physics. Single and multi-channel heat transfer performance was studied by using deionized water, as well as water-based nanofluids. The following results are concluded from our study:

1. For bended channels, the straight channel relations are reasonably valid at low Reynolds number with a 4% error in comparison to analytical results; however, at high Reynolds number, an increased error of up to a maximum to 17% can be seen due to an increase in pressure drop, non-uniformity of flow, and development of dean vortices.
2. Channels with less or equal height to that of width results in better convective heat transfer due to availability of more contact area with heated surface and increased space for asymmetric fluid to enter and leave bend. Moreover, secondary vortices phenomena are encountered due to the introduction of bend for channels of different hydraulic diameters, and it is found that the introduction of bend in channel significantly enhances the formation of dean vortices at Reynolds number greater than 500.
3. The increment in base temperature decreases the pressure drop due to a reduction in overall fluid viscosity and also decrease Nusselt number in comparison to low base temperature for the geometry of same parameter.
4. By utilizing performance parameters from the design point approach in Thermal Performance Factor (TPF) analysis, the performance of a single channel with 200 microns width is considered optimum and studied for height range from 35 to 300 microns for selection of multi-channel cross section.
5. For selected single channel, enhancement in thermal effectiveness is observed for both alumina and zirconia nanoparticles. At very low Reynolds number of around 100, nanofluids concentration showed no significant improvements. However, at very high Reynolds number, the TPF starts decreasing, representing dominance of pressure drop over convective heat transfer. Therefore, both alumina and zirconia with 1% and 3% concentration showed highest effectiveness at Re = 300. Maximum TPF value of 1.18 is achieved by zirconia with 1% concentration at Re = 600. It is seen that, by increasing nanoparticles concentration, pressure drop starts dominating at high Reynolds number.
6. For multi-channel with water as fluid, channel width is fixed at 200 μm and height is varied from 100 μm to 200 μm. Channel with 200 μm width and height showed a 33% to 50% increase in heat transfer for Reynolds number within a range of 100 to 900.
7. The introduction of nanofluids in 200 μm width and height multi-channel model showed enhancement in Nusselt number with an increase in Reynolds number from 100 to 600. The highest increment of 20% is observed in the case of zirconia, with 3% concentration at Re = 600. For 1% and 3% concentration of nanoparticles, zirconia outperformed alumina. Analysis of TPF showed that, after Re = 300, the slope of curve starts flattening in the case of 1% alumina and starts decreasing in the case of 1% and 3% zirconia and alumina 3% concentration, depicting increase in pressure loss with high concentration and Reynolds number due to increased viscosity.

## Figures and Tables

**Table 1:** Multi-channel model corresponding parameters.

Symbol	Dimension (μm)
l_1_	18,000
l_2_	4120
l_3_	2000
l_4_	1000
w_1_	440
w	200
w_s_	200
L	28,300
W	7000

**Table 2:** Temperature dependent fluid and fix solid properties.

Properties	Deionized Water [50,51]	Copper [52]
$\mu_{f}$ (Pa.s)	$0.0194-1.065\times 10^{-4}T+1.489\times 10^{-7}T^{2}$	--
$k_{nf}\;$(W/m.k)	$-0.829+0.0079T-1.04\times 10^{-5}T^{2}$	387.6
$C_{pf}$ (J/kg.k)	$5348-7.42T+1.17\times 10^{-2}T^{2}$	381
$\rho_{f}$ (kg/m^3^)	998.2	8978

**Table 3:** Nanoparticles’ properties.

Property	Alumina	Zirconia
$C_{np}$ (J/kgk)	880	418
$\;\rho_{np}$ (kg/m^3^)	3920	5600

**Table 4:** Single channel mesh characteristics.

Number of Elements	Pressure Drop (kPa)	Analytical [54]	% Error
11,664	122.3	146.5	16.5
22,350	132.6	146.5	9.5
59,486	143.4	146.5	2.1
181,000	149.3	146.5	1.8
384,813	151.5	146.5	3.3
1,958,264	154.2	146.5	5.2
2,844,375	154.6	146.5	5.5
4,373,200	154.7	146.5	5.5

**Table 5:** Studied channels with corresponding parameters.

Group	Width Range μm	Height Range μm	Temperature K	Number of Cases
G1	35–300	35–300	320	120
G2	35–300	35–300	325	120
G3	35–300	35–300	365	120
G4	35–300	35–300	370	120

**Table 6:** Correlations of Nusselt number based on curve fitting.

Channel	No Nanofluid	1% Alumina	3% Alumina	1% Zirconia	3% Zirconia
Straight	0.4396Re0.42	0.4058Re0.4533	0.3891Re0.4911	0.3973Re0.4686	0.4186Re0.5042
Bend	0.3177Re0.50	0.3109Re0.5227	0.3365Re0.5365	0.3138Re0.532	0.3715Re0.5442

**Table 7:** Cases for simple and modified multi-channel design.

Group	Width (μm)	Height (μm)	Temperature (K)
1	200	100–200	320
2	200	100–200	325
3	200	100–200	365
4	200	100–200	370

**Table 8:** Correlations of Nusselt number based on curve fitting.

Nanoparticles	0%	1%	3%
Alumina	3×10−7Re2+0.001Re+2.7808	−4×10−7Re2+0.0018Re+2.8255	−5×10−7Re2+0.0019Re+2.9944
Zirconia	3×10−7Re2+0.001Re+2.7808	−7×10−7Re2+0.002Re+2.8547	−7×10−7Re2+0.0024Re+3.2058

## Abbreviations

l_1_	Microchannel length (mm)
l_2_	Plenum Length (mm)
l_3_	Bend length (mm)
l_4_	Inlet/Outlet length (mm)
w_1_	Inlet/Outlet width (mm)
w	Width of microchannel (mm)
w_s_	Space between microchannels (mm)
L	Multi-Microchannel model length (mm)
L_c_	Microchannel model length (mm)
W	Microchannel model width (mm)
$\alpha_{\mathrm{ch}}$	Aspect ratio of microchannel
θ	Angle of plenum (mm)
Re	Reynolds Number
Nu	Nusselt Number
${\mathrm{Nu}}_{o}$	Benchmark Nusselt Number
f	Friction Factor
$f_{o}$	Benchmark Friction Factor
$D_{H}$	Hydraulic Diameter (mm)
$A_{\mathrm{ht}}$	Base area of microchannel (mm^2^)
Q	Total heat rate (J/s)
$\dot{m}$	Mass flow rate (kg/s)
$\dot{V}$	Volume flow rate (m^3^/s)
h	Height of microchannel (mm)
$v_{\mathrm{in}}$	Inlet velocity (m/s)
$P_{\mathrm{power}}$	Pump power (W)
$C_{p}$	Specific heat (J/kg.k)
ρ	Fluid density (kg/m^3^)
μ	Fluid Viscosity (Pa.s)
k	Thermal Conductivity (W/m.k)
T	Temperature (K)
$T_{W}$	Wall Temperature (K)
ΔT	Temperature Difference (K)
ΔP	Pressure Difference (Pa)
TPF	Thermal Performance Factor
Subscript	
f	Fluid (Water)
nf	Nanofluid
np	Nanoparticle
i	Inlet
o	Outlet
m	Mean
s	Source
so	Solid
